# Self-supported Ag(0) nanocatalyst derived from Ag(I)-based coordination polymer

**DOI:** 10.1038/s41598-025-20147-x

**Published:** 2025-09-23

**Authors:** Ahmad Baraka, Mohamed H. Alkordi, M. Gobara, M. Nasr Ettish, Mohamed Elbahy, Osama Abuzalat

**Affiliations:** 1 Department of Chemical Engineering, Military Technical College, Cairo, Egypt; 2 Center for Materials Science, Zewail City of Science and Technology, Giza, Egypt

**Keywords:** Silver nanoclusters, Coordination polymer, Self-Supported catalyst, Hydrogen peroxide decomposition, Radical-Mediated oxidation, Chemistry, Catalysis

## Abstract

A self-supported microsphere heterogeneous catalyst has been developed from a synthesized Ag(I)-imidazole coordination polymer (compound **1**), which was subsequently transformed into an Ag(0)-laden coordination polymer (compound **2**) through mild reduction with ascorbic acid. It is proposed that during the reduction process, a measurable fraction of silver ions (Ag(I)) undergo in-situ conversion into silver atoms (Ag(0)), forming uniformly distributed Ag(0) nano-clusters within the structural motif of **2**. Additionally, some Ag(0) atoms are suggested to retain the coordination environment of their precursor Ag(I) ions within the framework of **2**. The Ag(0)-laden microspheres in catalyst 2 exhibited exceptional zero-energy auto-catalytic decomposition of H_2_O_2_ at room temperature under the working conditions. This approach offers a straightforward synthesis route for a self-supported Ag catalyst with significantly enhanced atomic efficiency, catalyst accessibility, and durability, eliminating the need for conventional supports such as silica or alumina.

## Introduction

As catalytic processes continue to drive the vast majority of the chemical industry, heterogenous catalysis offers several advantages, including the ease of separating the catalyst from the bulk of reactants, the ability to regenerate the spent or deactivated catalyst, as well as enabling continuous process operation^1^. As the catalytic process in heterogeneous catalysis commences at the solid-liquid or solid-gas interface, maximizing the surface area of the catalyst is an important key towards boosting its activity and efficiency^2^. Size reduction of the catalyst, reaching down to micro- and nano-particles or even small clusters of catalytic sites, results in maximizing the surface-to-volume ratio of the nanocatalyst. While this approach is of great potential for impacting atom efficiency of catalysts, effective immobilizing such particles/clusters on suitable larger support is essential to avoid leaching into the reaction system, facilitate catalyst handling and processing, as well as avoid adverse health and environmental impacts associated with the miniscule size of the catalyst^3,4^. Though immobilizing catalysts on porous supports like silica and alumina is commonly explored, the self-supported catalyst is distinguished by size uniformity, homogeneous distribution of active sites, as well as better economics of operation^3^.

Metallic nanocatalysts, including gold, platinum, palladium, nickel, iron, and silver, demonstrate high catalytic performance in a large number of chemical processes, including, but not limited to, alkene hydrogenation, ammonia synthesis, hydrocarbon reformation, Fisher-Tropsch synthesis, methanol oxidation, among others^5^. A multitude of synthetic methods have been explored towards constructing tailorable supported nanocatalysts, including wet incipient impregnation, chemical vapor deposition (CVD) and atomic layer deposition (ALD), resulting in nanocatalysts deposited on solid support like zeolites^6^, silica^7^, alumina^8^, graphene^9,10^, and carbon nano tubes, allowing for controlled fabrication of nanoparticles with notable uniformity and control over the resulting size and shape of the supported nanoparticles^11^. Alternatively, a stable form of the nanocatalyst can be constructed without the need for solid support, known as a self-supported catalyst^12^.

For the past two decades, functional inorganic-organic hybrid frameworks have been extensively developed, utilizing ligands as Linkers and metal ions as nodes to form 1D, 2D, and 3D structures via coordination bonding. Due to their extended structures, these coordination polymer (CP) solids are typically insoluble in common reaction media, making them ideal candidates for heterogeneous catalysis when they incorporate appropriate catalytic functionalities. As a result, such materials can function as heterogeneous catalysts and serve as immobilized homogeneous catalysts without requiring external supports, effectively acting as self-supported catalysts^12^.

Recently, some interesting research has been published considering CP materials for different heterogeneous catalysis applications. A Bimetallic Mo–Zn infinite coordination polymer (ICP) nanoparticles were synthesized as a self-supported catalyst by post-modifying Zn-ICP with MoO_2_(acac)_2_. The resulting Mo–Zn-ICP showed high catalytic activity in olefin epoxidation, driven by cis-dioxo-Mo centers^13^. A single-site heterogeneous Pd(II)–NHC catalyst was developed on a Ru(II)–terpyridine-based coordination polymer. It efficiently promotes selective arene C–H mono-halogenation with high reusability. Control studies confirm the scaffold’s robustness and superior performance over the homogeneous analogue^14^. The sheet-like heterogeneous self-supported catalysts, dirhodium(ii) coordination polymers, were synthesized via ligand exchange using Rh_2_(TFA)_4_ or Rh_2_(OAc)_4_ with bdc linkers. The resulting Rh_2_-bdc frameworks retain the Rh–Rh bond and oxidation state. These self-supported catalysts exhibit high stability, reusability, and catalytic activity in styrene cyclopropanation^15^.

Self-supported coordination polymers capable of decomposing H_2_O_2_ are rarely reported. However, a notable study demonstrated the fabrication of self-supported Au–Pd@UiO-66-on-ZIF-L nanohybrids on flexible carbon cloth using a MOF-on-MOF strategy followed by Au electrodeposition. The resulting bimetallic system showed outstanding electrocatalytic activity for H_2_O_2_ reduction, achieving high sensitivity (390 µA mM‒1 cm‒2) and a low detection limit (21.2 nM)^16^.

Imidazole and its derivatives are prominent ligand candidates for CPs, metal-organic frameworks (MOFs), and zeolitic imidazolate frameworks (ZIFs) due to their two nitrogen donor coordination sites and form some of the most stable complexes among heterocyclic nitrogen ligands^17^. Silver(I)-imidazolate CP was first synthesized in 1995 and 1997, demonstrating a well-defined structure where single silver ions act as nodes^17,18^. Previous reports describe silver(I)-imidazole CP as a neutral Ag(I)-N bonding polymeric compound with silver(I) to imidazole in a 1:1 ratio^19^. IR analysis confirmed that imidazole coordinates Ag(I) as an anion rather than as a neutral ligand, while PXRD analysis revealed a polymeric chain structure with Linearly coordinated silver ions bridged by imidazolate Ligands, ensuring the formation of only cyclic, oligomeric species or 1D chains^19^.

In the present study, due to its ease of synthesis, the aforementioned simple motif, and well-characterized structure, Ag(I)-imidazole CP was selected for synthesis for the conversion into a self-supported zero-valent silver catalyst. Herein, we describe the synthesis of Ag(I)-imidazole CP, compound **1**, and its transformation into a self-supported microsphere heterogeneous catalyst, compound **2**, via post-synthetic mild reduction by ascorbic acid. Upon reduction, it is suggested that Ag(0) nano-clusters were homogenously formed and distributed within the compound **2** motif, offering promising catalytic potential. To investigate such catalysis, the decomposition of H_2_O_2_ was performed, and the rate is given and discussed. Also, a proposal for the decomposition mechanism is properly given.

## Experimental work

### Materials

Imidazole (C₃H₄N₂, 99%, Sigma-Aldrich), ammonia solution (NH₃, 35%, Fisher Scientific), and silver nitrate (AgNO_3_, 99%, Sigma-Aldrich) were used as received without further purification. L-Ascorbic acid (C_6_H_8_O_6_, ≥ 99%, Sigma-Aldrich) was used for the reduction process. (Hydrogen peroxide (H_2_O_2_, 50% commercial). DI water was used throughout the synthesis. All chemicals were of analytical grade and purchased from commercial suppliers.

### Synthesis of Ag-Imidazolate coordination polymer

To synthesize Ag-Imidazolate, compound **1**, a beaker (100 ml) was loaded with imidazole (0.68 g/20 ml DI-water) and ammonia solution (35%, 1.3 ml). The solution was stirred on a hot plate adjusted at 90 °C for about five minutes to guarantee stabilized deprotonation of N-pyrrole of imidazole. Silver nitrate solution (AgNO_3_, 1.7 g/10 ml DI-water) was then added to the stirred ammoniated imidazolate solution, where immediately a dense white suspended cloud was formed occupying the whole solution volume. Stirring was allowed to continue for about an extra thirty minutes at the same temperature, where the white cloud volume was continually converting into particle aggregates. Stirring and heating were stopped, and the solution was left for complete settling down of the white product, compound **1**. After cooling down, **1** powder was then separated by simple filtration from the mother solution and then washed 3 times using adequate amounts of DI-water. The wet solid compound **1** was then allowed to dry at ambient temperature. The measured yield of **1** was about 34%.

The novel compound **2** was thereafter prepared from **1** by reduction using L-ascorbic acid. A certain dried amount of **1** was treated by a low concentration of L-ascorbic acid aqueous solution with shaking for about 5 min at ambient temperature, producing compound **2**. The powder of **2** was quickly separated from reducing solutions by simple filtration and then washed 3 times using adequate amounts of DI water. The wet solid reduced samples were then allowed to dry at ambient temperature. SEM/EDX (Zeiss EVO-10 microscopy), FTIR (KBr pellet method using Jasco FT/IR 4100), Powder X-ray diffraction (PXRD, Shimadzu XD-l), and XPS Thermo Fisher Scientific-USA, monochromatic Al-K_α_, 1486.7 eV) were applied to characterize compounds **1** and **2.**

To examine the catalytic decomposition of H_2_O_2_ by **2**, a H_2_O_2_ solution (5000 ppm) was used as the starting material. The reagents included 0.1 N potassium permanganate (KMnO_4_) solution, 1 N sulfuric acid (H_2_SO_4_), and DI water. A known volume (50 ml) of the 5000 ppm H_2_O_2_ solution was transferred into a reaction vessel. A pre-determined quantity of the catalyst (10, 20, 30 mg) was introduced, and the reaction was allowed to proceed with decomposition. Every 10 min, 10 ml of the reaction mixture was transferred to a 100 ml conical flask. To this, 10 ml of 1 N H_2_SO_4_ was added to acidify the solution, ensuring complete reaction of H_2_O_2_ with KMnO_4_. A burette (50 ml) was filled with standardized 0.1 N KMnO_4_ solution. The acidified H_2_O_2_ solution was titrated by the gradual addition of KMnO_4_ with constant swirling until a persistent pink colour appeared, indicating the endpoint. The volume of KMnO_4_ solution used in the titration was recorded. The residual H_2_O_2_ concentration was calculated using the equation:

$$\begin{gathered} {C_{{\mathbf{H2O2}}}}={V_{{\mathbf{KMnO4}}}} \times {N_{{\mathbf{KMnO4}}}} \times {\mathbf{17}}/{V_{{\mathbf{sample}}}} \hfill \\ \hfill \\ \end{gathered}$$

Where C_H2O2_ is the concentration of residual H_2_O_2_ (mg/l), *V*_KMnO4_ is the volume of KMnO_4_ consumed (ml), *N*_KMnO4_ is the normality of KMnO_4_ solution (N), and *V*_sample_ is the volume of sample taken (50 ml). Titration was performed in triplicate to ensure accuracy and reproducibility. The obtained values were compared with the initial 5000 ppm concentration to determine the extent of catalysis.

To evaluate the silver retention of compound **2** within its matrix, 0.15 g of the material was immersed in 50 mL of deionized water containing 10% H_2_O_2_. The sample was left undisturbed for 6 h to allow potential leaching of Ag(I) ions, after which inductively coupled plasma (ICP) analysis was conducted.

The catalytic degradation of methylene blue (MB) was conducted to evaluate the oxidative performance of the self-supported Ag(0) catalyst, compound **2**. In a typical experiment, 50 mL of an aqueous MB solution (10 mg/l) was prepared and transferred into a 100 mL beaker. To this, 30 mg of the Ag(0) catalyst was added under constant magnetic stirring at room temperature. Subsequently, 1 mL of freshly prepared H_2_O_2_ (5 wt%) was introduced to initiate the reaction. The reaction mixture was kept under continuous stirring, and aliquots (3 mL) were withdrawn at regular time intervals (0, 5, 10, 15, 20, and 30 min). Each aliquot was immediately centrifuged at 5000 rpm for 5 min to remove catalyst particles. The clear supernatant was analyzed using UV–Vis spectroscopy by monitoring the absorbance at 664 nm, the characteristic absorption peak of MB. Control experiments were also performed in the absence of either the catalyst or H_2_O_2_ to confirm the role of each component in the degradation process. Additionally, radical scavenger experiments were conducted to investigate the active species involved in the catalytic mechanism.

## Results and discussion

### Characterization of compounds

**Fig. 1:**
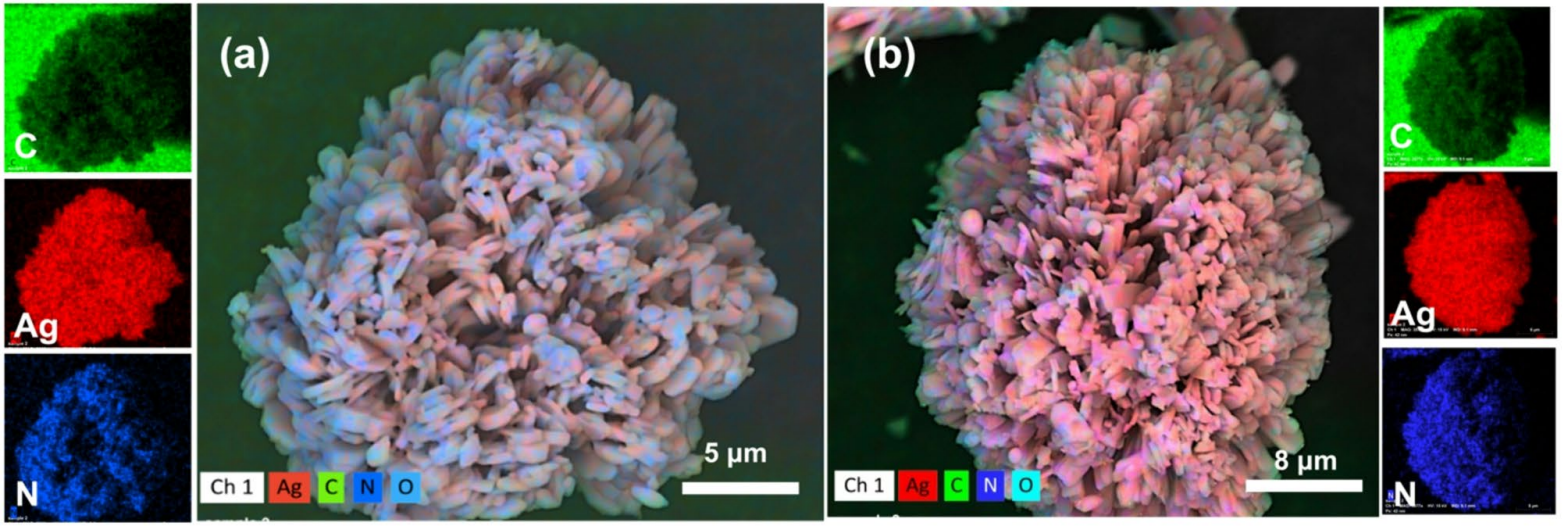
SEM images of (**a**) compound **1** and (**b**) after mild treatment with ascorbic acid as reducing agent to form compound **2**, with inserts showing EDX mapping of C, N, and Ag elements

The hydrothermal synthesis of Ag(I)-imidazolate coordination polymer, **1**, utilized the affinity of the soft Lewis acid Ag(I) and soft Lewis base (imidazole), where Ag-N coordination interactions are expected to result in an extended network solid insoluble in the reaction medium. The scanning electron microscopy (SEM) images of **1** and its reduced form, **2**, are shown in Fig. 1. Due to favourable solid-solid interactions, the resulting platelets of **1** self-assembled into microspheres of regular and uniform shape and size, Fig. 1**(a)**. The Ag(I) ion within the coordinated structure in the microspheres of **1** can easily be reduced upon treatment with an aqueous solution of ascorbic acid, resulting in the reduced form maintaining the same size and morphology, **2** Fig. 1**(b)**. Elemental mapping of C, N, and Ag was also recorded using energy dispersive X-ray analysis (EDX), where the elemental maps of the three selected elements trace the SEM images of **1** and **2**, indicating uniformity of the Ag element in both samples, inserts in Fig. 1.

From Fig. 1(b), no Ag(0) micro-aggregates or micro-clusters are observed on the particle surface of **2**. This suggests that no significant leaching of Ag(I) from the structure of **1** occurred during the reduction process^20^. Consequently, the reduced silver did not materialize as surface-deposited Ag(0), supporting the feasibility of an alternative reduction mechanism. Specifically, the reduction likely proceeded via in-situ conversion of Ag(I) nodes within the pristine structural matrix, preserving their original coordination environment, and/or through the formation of internal Ag(0) nano-clusters within the framework of **2**. In either case, the well-distributed metallic silver within **2** enhances its potential as a catalytic material for H₂O₂ reduction.

**Fig. 2:**
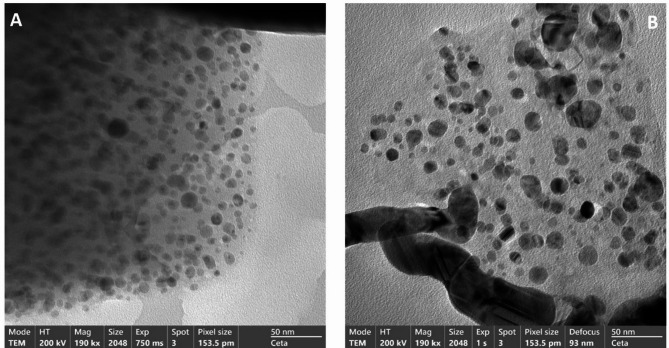
HRTEM image of (**A**) compound **1**, and (**B**) compound **2**

Figure 2 shows HRTEM images of compounds **1** and **2**. Image **1 (**pristine CP) shows a uniform distribution of dark spherical spots of similar contrast, which indicates these spots are of a specific moiety type. These spots are likely Ag(I)-rich domains or possibly lightly crystalline **1** regions. Also, the absence of lattice fringes suggests limited crystallinity or high dispersion of Ag(I) in the polymeric matrix. The soft contrast is consistent with apparently Ag(I) ions uniformly distributed/coordinated within the framework. On the other hand, the image of **2 (**reduced coordination polymer) shows strong contrast with larger and darker spots. Clearly, some of these spots show obvious crystalline boundaries or lattice planes, which strongly indicate the formation of metallic Ag nanoparticles, Ag(0). The increased darkness strongly suggests partial reduction of Ag(I) to Ag(0). As a conclusion, the changes between the two images suggest that the reducing agent induced partial reduction of Ag(I) within the coordination polymer matrix to metallic Ag nanoparticles. This supports the proposal that the coordination polymer acts as an in-matrix for metallic nanocluster formation.

**Fig. 3:**
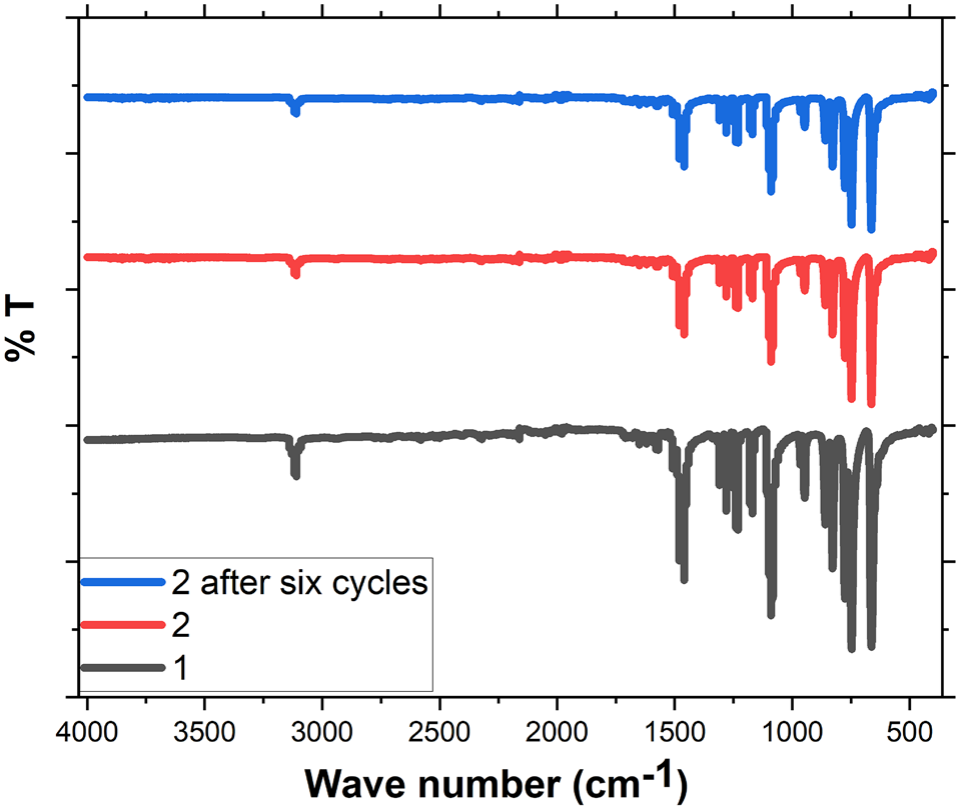
FTIR spectra of **1** (black line), **2** (red line) indicate the maintained structure of **1** upon surface reduction with ascorbic acid, and (blue) after 6 cycles of H2O2 decomposition

FTIR analysis was employed to further characterize compounds **1** and **2** (Fig. 3**)**. Both compounds exhibited characteristic absorption bands at 1461 and 1475 cm^–1^, corresponding to C = N stretching, along with a sharp absorption at 3113 cm^–1^, characteristic of sp^2^ C–H stretching within the imidazolate ring. Notably, the FTIR spectra of the pristine compound **1** and the mildly reduced compound **2** are nearly identical, suggesting that the overall network of the pristine solid remains intact upon mild reduction. This preservation is likely attributed to minimal alterations in the coordination environment of the Ag centers, with the reduction of Ag(I) ions occurring primarily at the crystal surface rather than penetrating deeply into the bulk material. The compound 2 also shows good stability after 6 cycles of H_2_O_2_ decomposition.

**Fig. 4:**
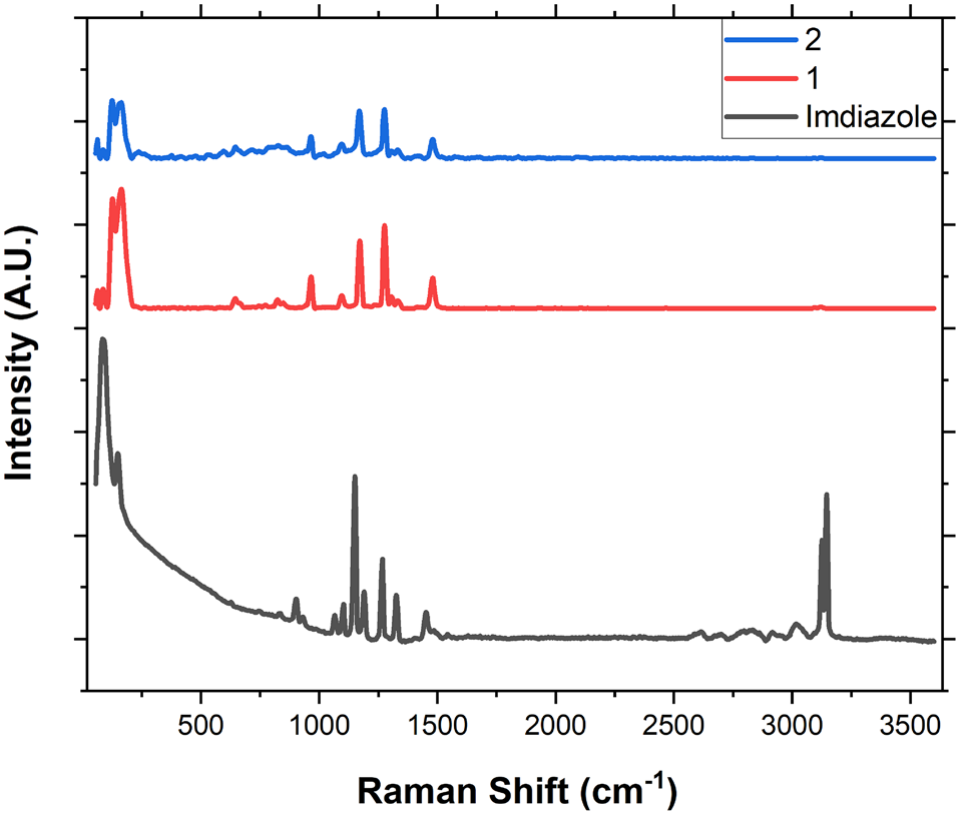
Raman spectra of imidazole, compound **1**, and compound **2**

Raman is a powerful tool to investigate CPs and MOFs, and hence was applied to compounds **1** and **2**^21–23^. Figure 4 shows Raman spectra of imidazole and compounds **1** and **2**. Regarding the crystallinity range, (0–300 cm^–1^), the imidazole spectrum has one strong peak at 121.5 cm^–1^ and one weak peak at 139.5 cm^–1^. The compound **1** spectrum shows two strong peaks at 124.5 and 165 cm^–1^ which indicates successful synthesis of compound **1** from imidazole. The fingerprint range (300–1800 cm^–1^) also indicates the successful synthesis of compound **1** from imidazole while conserving the imidazole structure. This is clear from the observed shifts of all peaks of imidazole in this range upon forming compound **1**^24,25^. The extended range (1800–3500 cm^–1^), more obviously, confirms the successful synthesis of compound **1** from imidazole. The characteristic strong peaks of imidazole at 3129 and 3146 cm^–1^ completely vanished upon formation of compound **1**. Finally, the spectrum of compound **2** is very similar and almost identical to that of compound **1**, indicating that compound **1** withstands stable ascorbic acid reducing treatment, preserving structure and functionalities upon conversion into compound **2**.

**Fig. 5:**
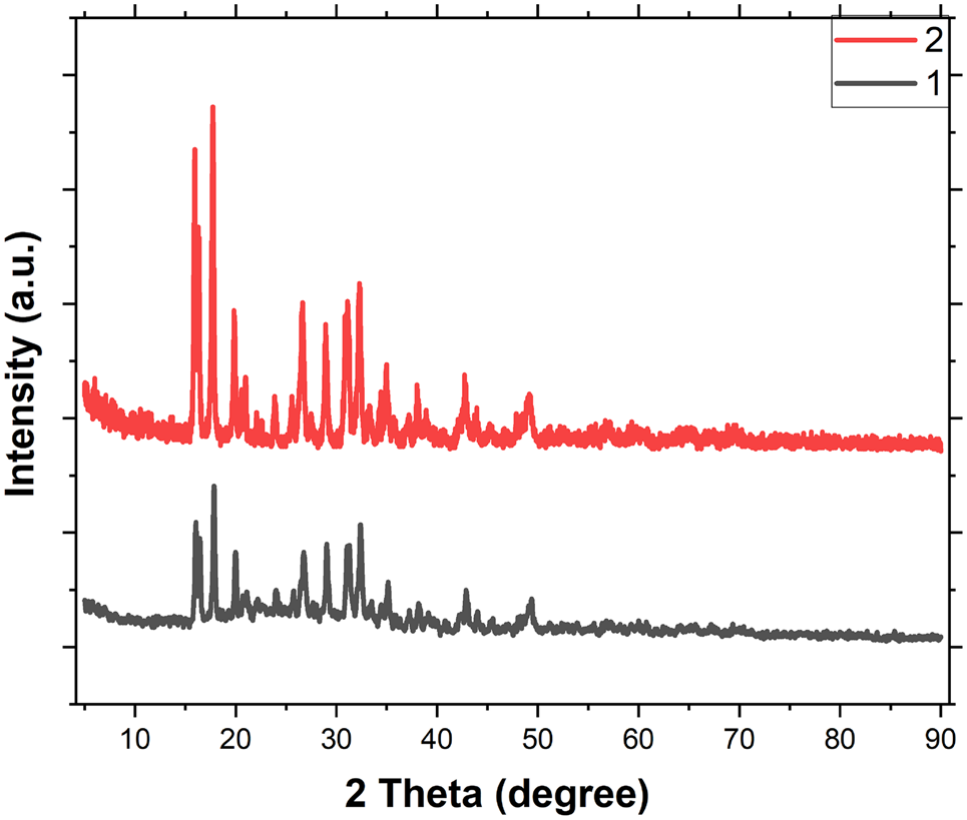
XRD patterns of compounds **1** and **2** indicate maintained crystallinity and absence of diffraction peaks characteristic of Ag particles

Figure 5 presents the XRD patterns of compounds **1** and **2**, both exhibiting sharp peaks indicative of well-defined crystalline structures. The remarkable similarity between the two patterns suggests that the reduction treatment did not disrupt the ordered arrangement of silver atoms and imidazole moieties within the material’s matrix. Furthermore, the absence of diffraction peaks corresponding to metallic Ag particles in the pattern of compound **2** implies that the reduction process occurred in highly localized regions, likely confined to the silver coordination nodes and/or the formation of Ag(0) nano-clusters within the structure.

**Fig. 6:**
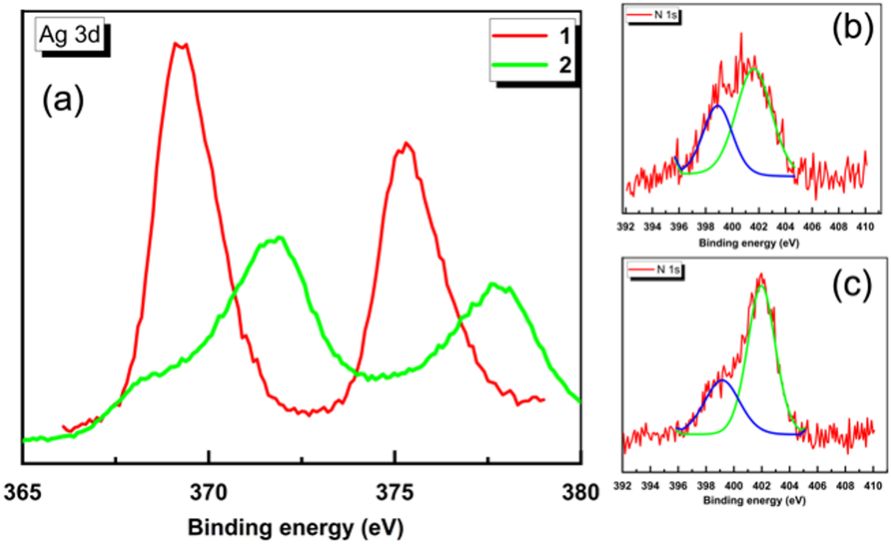
XPS analysis of **1** and **2**, (**a**) Ag 3 d line scan indicating reduction of Ag(I) in **1** to Ag(0) in **2**, (**b**) N1s line in **1** demonstrating two nearly equivalent nitrogen environments, while (**c**) the N1s line scan in **2** demonstrating pronounced pyrrolic N environment upon surface treatment with reducing agent

All the above analyses suggest that compound **2** kept the morphological and topological natures of compound **1** upon reduction. The only possible change is the reduction of Ag(I) ions into Ag(0) atoms, preserving the coordination position and environment. The X-ray photoelectron spectroscopy may help support this proposal, and XPS analysis of **1** and **2** is shown in Fig. 6. The Ag 3 d line spectra indicated reduction of Ag(I) to Ag(0), the Ag 3d_5/2_ Line shift from 369.27 eV to 371.71 eV, a shift of + 2.44 eV, which is significantly greater than upshifts commonly recorded for the change of oxidation state. Additionally, the peak width increased upon reduction of Ag(I) to Ag(0). These two observations pointed out to reduction of Ag(I) to Ag(0) as well as the concomitant formation of ultra-small cluster size as shown in Fig. 6A^26^.

For more explanation, the observed + 2.44 eV upshift in the Ag 3d_5_/_2_ XPS signal, from 369.27 eV in the pristine CP to 371.71 eV after mild reduction with ascorbic acid, deviates significantly from the conventional downshift typically associated with the reduction of Ag(I) to metallic Ag(0). This unusual behaviour can be rationalized by considering that both the initial and reduced CPs consist of nanoscale silver clusters embedded within an imidazole-based coordination polymer matrix^27^. For compound **1**, Ag(I) ions are strongly coordinated to nitrogen donors of the imidazole ligands, which can induce abnormally high binding energies due to electron withdrawal and poor core-hole screening^28^. Upon reduction, metallic Ag(0) clusters are formed, but remain confined within the coordination polymer matrix as shown in Fig. 6A. These Ag(0) nanoclusters do not behave like bulk metallic silver; instead, they are subject to quantum confinement and limited electronic screening. These effects can lead to increased binding energies, occasionally even exceeding those of their oxidized counterparts. Therefore, the unexpected upshift is consistent with the formation of ultrasmall, matrix-confined Ag(0) clusters materials matrix.

As previously outlined, the screening of core holes in ultra-small metal nano-clusters becomes limited, which results in an upshift of core-level binding energy^26,29–31^. In agreement with this proposed assignment, the N1s lines recorded for **1** and **2**, Fig. 6b and c, respectively, indicated two distinctive chemical environments ascribed for pyridine-like N (399 eV) and pyrrole-like N (401.9 eV), with the later more pronounced in **2**, indicating cleavage of some Ag-N bonds upon Ag(I) reduction with ascorbic acid.

### Decomposition of H_2_O_2_ by compound 2

**Fig. 7:**
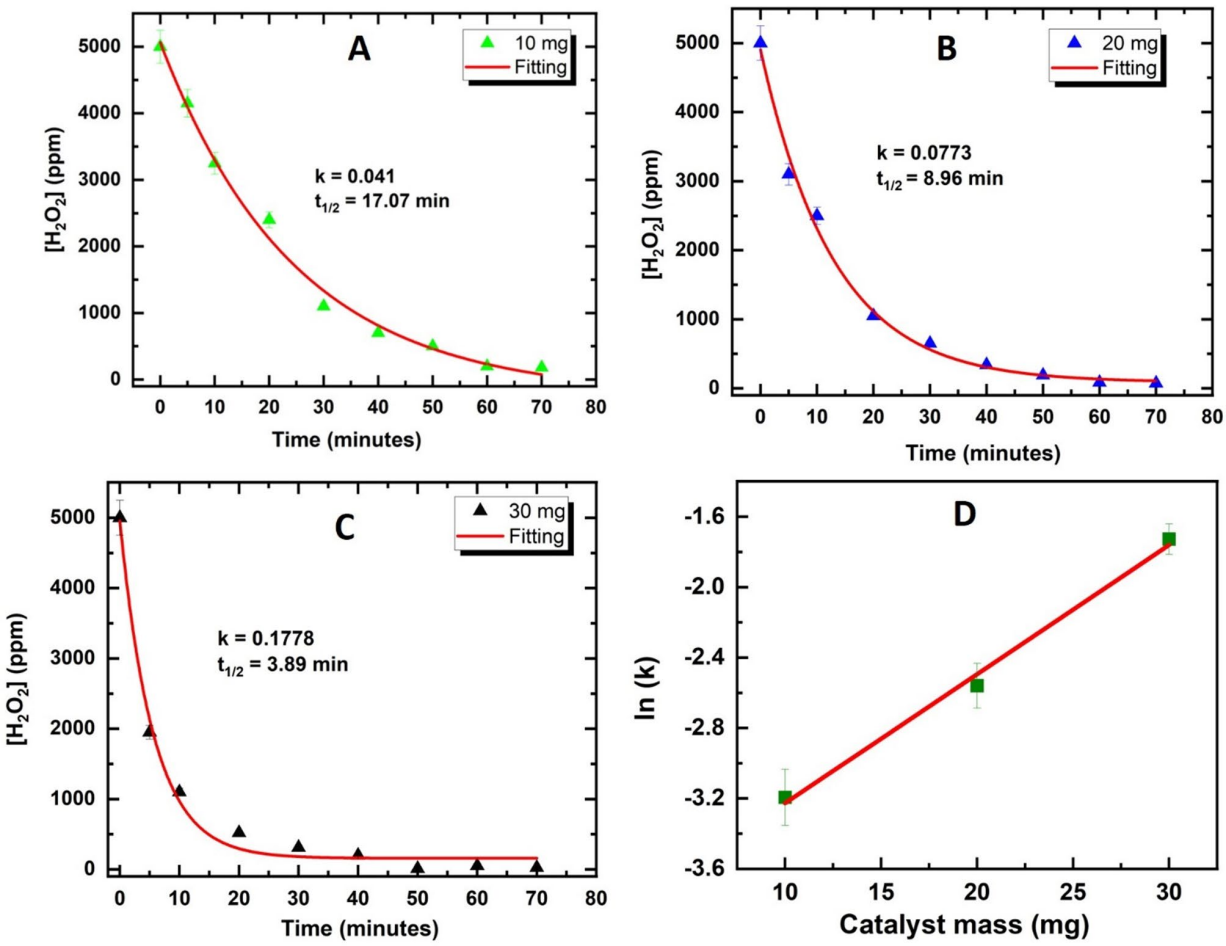
Catalytic decomposition of H_2_O_2_ utilizing compound **2** with different catalyst loading (**A**,** B**,** C**), and (**D**) linear fit with different mass loading

To probe the catalytic activity of the self-supported Ag(0) catalyst, different masses of **2** were introduced into H_2_O_2_ solution, and the remaining concentration of H_2_O_2_ was monitored through iodometric titration^32^, Fig. 7**(D)** demonstrating pseudo-first-order kinetics. A plot of initial reaction rate versus catalyst loading demonstrated a Linear behaviour, characteristic of surface-catalyzed reactions. Three different experiments utilizing catalyst loading of 10, 20, and 30 mg into 5,000 ppm H_2_O_2_ solution were utilized, where the three reactions converged to full decomposition of H_2_O_2_ at 60, 50, and 40 min, respectively, demonstrating the effective nature of the self-supported Ag(0) catalyst as shown in Fig. 7 (A, B, C). As a control experiment, compound **1**, containing only the Ag(I) species, did not produce notable activity towards decomposition of H_2_O_2_ even after 70 min of contact time with H_2_O_2_, confirming the catalytic activity of supported Ag(0) species in **2**.

ICP analysis of the leachate revealed an Ag(I) concentration of 48.3 ppm, corresponding to a total leached amount of 2.42 mg in 50 mL. Considering the chemical yield of the sample preparation (34%), the estimated total silver content in the sample was 110 mg. Therefore, the percentage of silver leached over 6 h was calculated to be 2.2%. This low percentage suggests recognized stability of compound **2** against H_2_O_2_.

**Fig. 8:**
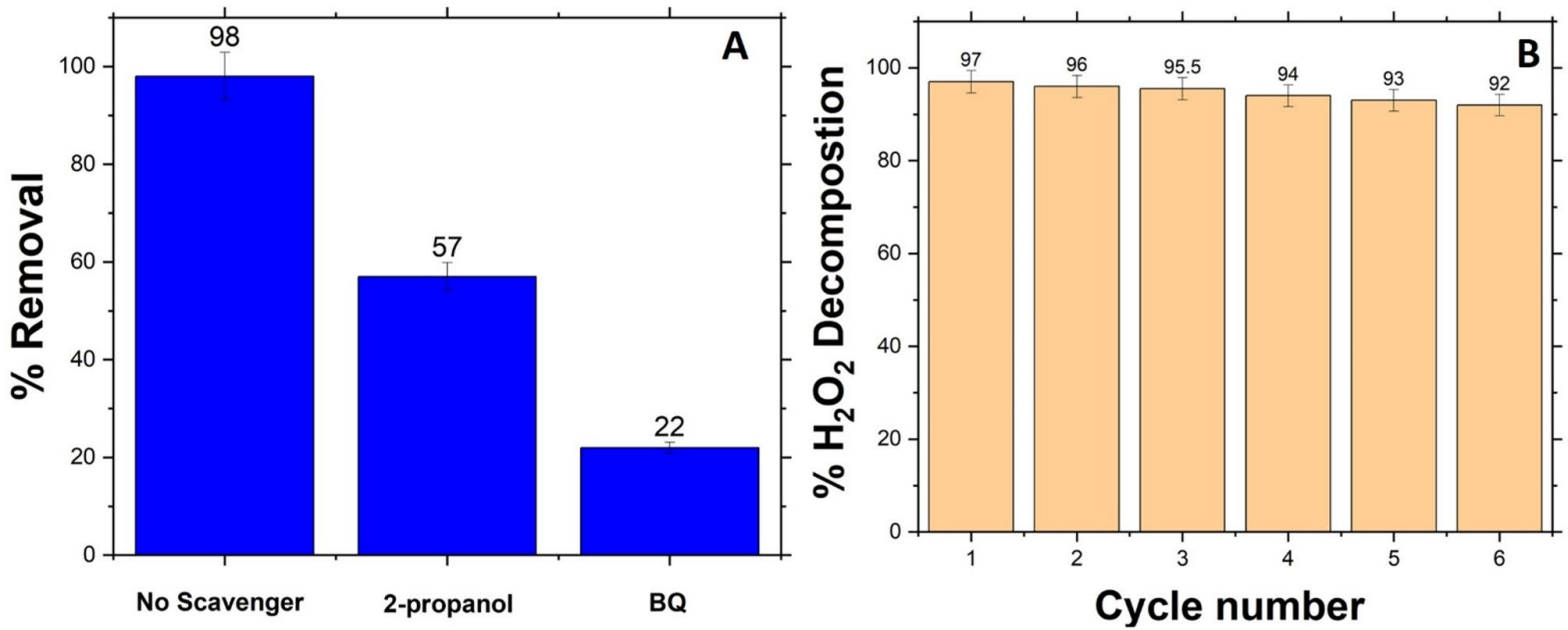
(**A**) Effect of radical scavengers on H₂O₂ removal efficiency: 2-propanol (^•^OH scavenger) and benzoquinone (O₂⁻^•^ and HO_2_^•^ scavenger), confirming the involvement of hydroxyl and superoxide radicals in the catalytic mechanism, and (B) Recyclability of the self-supported Ag(0) catalyst for H_2_O_2_ decomposition over six consecutive cycles, demonstrating high catalytic stability

The catalytic performance of the material was evaluated in terms of both reactive species involvement and reusability, which are critical parameters for practical applications. To elucidate the underlying degradation mechanism of H_2_O_2_, scavenger experiments were conducted to identify the key reactive oxygen species (ROS) involved. As shown in Fig. 8A, the introduction of 2-propanol, a specific scavenger for hydroxyl radicals (^•^OH), significantly reduced the MB removal efficiency from 98% (in the absence of any scavenger) to 57%. This result indicates that ^•^OH plays a major role in the decomposition process catalyzed by compound **2**. Furthermore, when benzoquinone (BQ), a known superoxide radical (O_2_⁻^•^ and HO_2_^•^) scavenger, was added, the removal efficiency decreased even further to 22%. This substantial inhibition confirms the participation of both O_2_⁻^•^ and HO_2_^•^ species as another dominant ROS in the system. These findings suggest a synergistic mechanism involving both hydroxyl and superoxide radicals in the catalytic breakdown of MB^32^.

The prominent decline in performance upon scavenger addition provides strong evidence for a radical-mediated degradation pathway, facilitated by electron transfer processes on the surface of Ag(0) nanoclusters. The metallic silver likely acts as an electron relay center, activating H_2_O_2_ and promoting the formation of ROS species.

In addition to its high catalytic activity, the recyclability of the catalyst was examined over six successive cycles, as shown in Fig. 8B. The catalyst maintained excellent stability, with H_2_O_2_ decomposition efficiency decreasing only slightly from 97% in the first cycle to 92% by the sixth. This marginal drop indicates good structural integrity and durability of the catalyst under repeated use. The retained performance over multiple cycles designates the robust nature of the self-supported Ag(0) system, compound **2**. No significant agglomeration or deactivation was observed, suggesting that the active silver sites remain accessible and functional throughout the reaction runs.

To assess the competitiveness of compound **2** relative to other similar catalysts, Table 1 presents a comparison based on their rate constants. A key advantage of compound **2** lies in its ability to achieve efficient catalysis using a nominal amount of active sites, Ag(0), in contrast to the higher metal loadings typically required by other materials.

**Table 1:** Comparison of the catalytic decomposition of hydrogen peroxide by compound 1 with others in the literature.

Material	k (min^−1^)	Ref.
HfO_2_	2.6 × 10^−2^	^33^
FeOOH	2.1 × 10^−2^	^34^
GAC1a	5.9 × 10^−4^	^35^
Ag(0)-IM CP	4.1 × 10^−2^	This work

### Proposed mechanism of H_2_O_2_ decomposition over compound 2

**Fig. 9:**
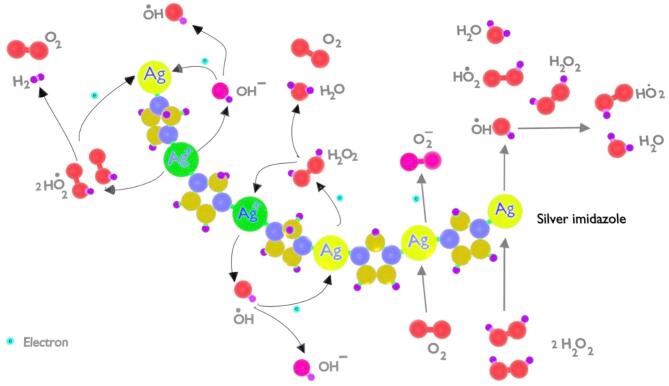
Schematic illustration of the proposed mechanism of H_2_O_2_ Decomposition over Compound **2**

The decomposition of H₂O₂ by compound 2 is driven by the interaction of Ag(I) and Ag(0) sites with H_2_O_2_. Ag(I) binds H_2_O_2_ through oxygen atoms, weakening the O–O bond. Ag(0) adsorbs H₂O₂ via electronic interactions and transfers electrons, generating ^•^OH and HO_2_^•^ radicals that desorb from the surface^36^. Additionally, superoxide anions (O_2_^•^⁻) are also generated. The highly reactive ^•^OH radicals further interact with non-adsorbed H_2_O_2_ molecules, leading to further decomposition^37^ as shown in Fig. 9 :

$$\begin{array}{*{20}{l}} {2{H_{2}}{O_2}{\text{ }} - {\text{ }}Ag\left( 0 \right){\text{ }}{ \to ^ \bullet }OH\,+\,H{O_2}^{ \bullet }+{\text{ }}{H_2}O} \\ {{O_2}+{\text{ }}Ag\left( 0 \right){\text{ }} \to {\text{ }}{O_2}^{{ \bullet - }}{\text{ }}+{\text{ }}Ag\left( I \right)} \\ {^{ \bullet }OH\,+\,{H_2}{O_2}{\text{ }} \to {\text{ }}{H_2}O\,+\,H{O_2}^{ \bullet }} \end{array}$$

Ag(0)/Ag(I) redox cycle should also occur^38^:

$$\begin{array}{*{20}{l}} {Ag\left( 0 \right)\,+\,{H_2}{O_2}{\text{ }} \to {\text{ }}Ag\left( I \right)\,+\,{H_2}O{\text{ }}+{\text{ }}\raise.5ex\hbox{$\scriptstyle 1$}\kern-.1em/ \kern-.15em\lower.25ex\hbox{$\scriptstyle 2$} {\text{ }}{O_2}} \\ {Ag\left( 0 \right)\,+\,{O_2}^{{ \bullet - }} \to {\text{ }}Ag\left( 0 \right){*^ - }+{\text{ }}{O_2}} \\ {Ag\left( I \right)\,+\,Ag\left( 0 \right){*^ - } \to {\text{ }}2Ag\left( 0 \right)} \\ {Ag\left( I \right){\text{ }}{+^ \bullet }OH{\text{ }} \to {\text{ }}Ag\left( 0 \right)\,+\,O{H^ - }} \\ {Ag\left( I \right)\,+\,2H{O_2}^{ \bullet } \to Ag\left( 0 \right)\,+\,2{O_2}\,+\,{H_2}} \\ {Ag\left( I \right)\,+\,O{H^ - } \to {\text{ }}Ag\left( 0 \right){\text{ }}{+^ \bullet }O{H^{}}} \end{array}$$

Further, Ag(I) can stabilize reaction intermediates, including ^•^OH and HO_2_^•^, acting as a Lewis acid to coordinate with H_2_O_2_ and its decomposition products. This coordination influences the breakdown of H_2_O_2_ into water and oxygen. The overall reaction for H_2_O_2_ decomposition in the presence of compound **2** is summarized as follows:

$${H_2}{O_2}{\text{ }}+{\text{ }}Ag\left( I \right){\text{ }} - {\text{ }}Ag\left( 0 \right)/Ag\left( I \right) \to {\text{ }}{H_2}O{\text{ }}+{\text{ }}\raise.5ex\hbox{$\scriptstyle 1$}\kern-.1em/ \kern-.15em\lower.25ex\hbox{$\scriptstyle 2$} {O_2}$$

A catalytic cycle is proposed, wherein Ag(I) is reduced back to Ag(0) by the decomposed H_2_O_2_​ or radical species, allowing continuous recycling of silver sites^38^.

## Conclusion

This work demonstrated the generation of highly efficient self-supported Ag(0) nanocatalysts from a simple structured Ag(I)-based coordination polymer through mild reduction. Ag(0) moieties within the coordination polymer should be nano-clusters or even Ag(0) nodded atoms. For both cases, metallic Ag is well distributed within the coordination polymer motif, giving a high chance for effective catalysis performance, especially when the coordination polymer is of micro-size with a featured platelet surface. Hence, the large library of previously reported coordination polymers or Metal-Organic Frameworks is an excellent candidate for this approach, where pre-designed catalytic metallic sites can be readily generated from ligand-metal coordination complex at the solid-liquid interface in a mild reducing solution.

## Data Availability

The datasets used and/or analysed during the current study available from the corresponding author Osama Abuzalat on reasonable request.
